# Exploring computational approaches to design mRNA Vaccine against vaccinia and Mpox viruses

**DOI:** 10.1002/iid3.1360

**Published:** 2024-08-16

**Authors:** Elijah K. Oladipo, Olanrewaju D. Oyelakin, Abdulsamad O. Aiyelabegan, Elizabeth O. Olajide, Victoria O. Olatayo, Kaothar P. Owolabi, Yewande B. Shittu, Rhoda O. Olugbodi, Hezekiah A. Ajala, Raji A. Rukayat, Deborah O. Olayiwola, Boluwatife A. Irewolede, Esther M. Jimah, Julius K. Oloke, Taiwo O. Ojo, Olumide F. Ajani, Bamidele A. Iwalokun, Olatunji M. Kolawole, Olumuyiwa E. Ariyo, Daniel A. Adediran, Seun E. Olufemi, Helen Onyeaka

**Affiliations:** ^1^ Division of Vaccine Design and Development Helix Biogen Institute Ogbomoso Oyo State Nigeria; ^2^ Laboratory of Molecular Biology, Immunology and Bioinformatics, Department of Microbiology Adeleke University Ede Osun State Nigeria; ^3^ Molecular Biology and Biotechnology Department Nigeria Institute of Medical Research Lagos Nigeria; ^4^ Department of Natural Sciences Precious Cornerstone University Ibadan Oyo State Nigeria; ^5^ African Centre for Disease Control HQ Addis Ababa Ethiopia; ^6^ Department of Microbiology University of Ilorin Ilorin Kwara State Nigeria; ^7^ Department of Medicine, Infectious Disease and Tropical Medicine Unit Federal Teaching Hospital Ido Ekiti Ekiti State Nigeria; ^8^ Department of Biochemistry Ladoke Akintola University of Technology Ogbomoso Oyo State Nigeria; ^9^ School of Chemical Engineering University of Birmingham Birmingham UK

**Keywords:** immunoinformatics, Mpox virus, mRNA vaccine, Pox viruses, vaccine design, vaccinia virus

## Abstract

**Background:**

Messenger RNA (mRNA) vaccines emerged as a powerful tool in the fight against infections. Unlike traditional vaccines, this unique type of vaccine elicits robust and persistent innate and humoral immune response with a unique host cell‐mediated pathogen gene expression and antigen presentation.

**Methods:**

This offers a novel approach to combat poxviridae infections. From the genome of vaccinia and Mpox viruses, three key genes (E8L, E7R, and H3L) responsible for virus attachment and virulence were selected and employed for designing the candidate mRNA vaccine against vaccinia and Mpox viral infection. Various bioinformatics tools were employed to generate (B cell, CTL, and HTL) epitopes, of which 28 antigenic and immunogenic epitopes were selected and are linked to form the mRNA vaccine construct. Additional components, including a 5′ cap, 5′ UTR, adjuvant, 3′ UTR, and poly(A) tail, were incorporated to enhance stability and effectiveness. Safety measures such as testing for human homology and in silico immune simulations were implemented to avoid autoimmunity and to mimics the immune response of human host to the designed mRNA vaccine, respectively. The mRNA vaccine's binding affinity was evaluated by docking it with TLR‐2, TLR‐3, TLR‐4, and TLR‐9 receptors which are subsequently followed by molecular dynamics simulations for the highest binding one to predict the stability of the binding complex.

**Results:**

With a 73% population coverage, the mRNA vaccine looks promising, boasting a molecular weight of 198 kDa and a molecular formula of C_8901_H_13609_N_2431_O_2611_S_48_ and it is said to be antigenic, nontoxic and nonallergic, making it safe and effective in preventing infections with Mpox and vaccinia viruses, in comparison with other insilico‐designed vaccine for vaccinia and Mpox viruses.

**Conclusions:**

However, further validation through in vivo and in vitro techniques is underway to fully assess its potential.

## Graphical abstract

The article presents the development of a novel messenger RNA (mRNA) vaccine targeting poxviridae infections. Through bioinformatics analysis and molecular simulations, the vaccine construct containing 28 antigenic epitopes was designed, with safety measures to avoid autoimmunity. The mRNA vaccine demonstrated promise, showing potential for preventing infections with Mpox and vaccinia viruses.

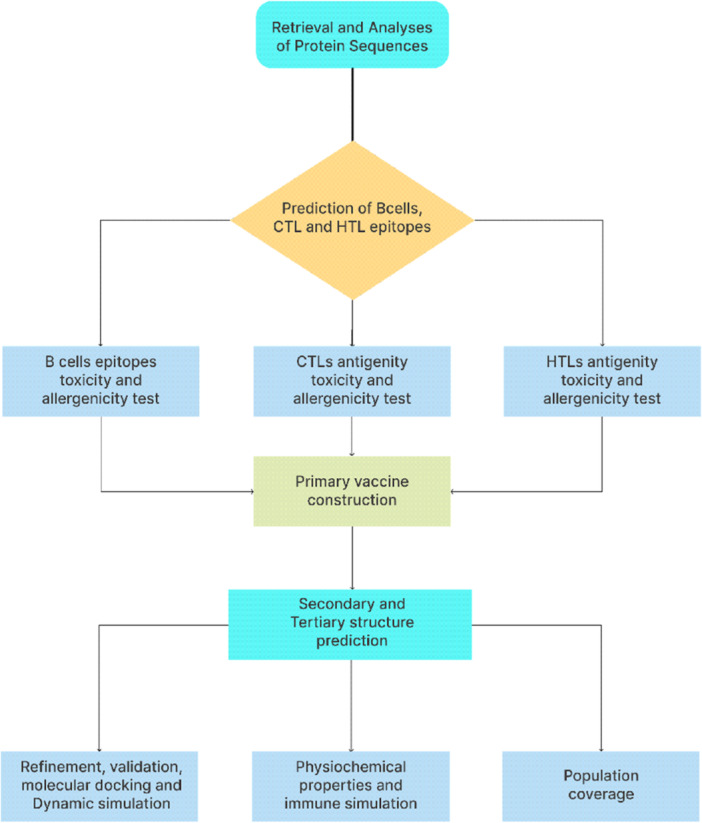

## 1 INTRODUCTION

The emergence of Mpox virus disease has been a concern for public health authorities, even as the world is still grappling with the outbreak of coronavirus disease (COVID‐19).^1^ Mpox virus, a member of the orthopoxvirus genus, is a double‐stranded DNA virus that encompasses variola, cowpox, and vaccinia viruses.^2^ In the pursuit of eradicating smallpox in the Democratic Republic of the Congo, Mpox virus emerged as a distinct human infection.^3^, ^4^ It was discovered in a patient reallyexhibiting symptoms resembling smallpox.^5^

Most of the clinical characteristics of human Mpox infection are similar to those of smallpox and studies have revealed that the smallpox vaccine offers cross‐protection against other orthopoxvirus species, including Mpox virus (MPXV).^6^, ^7^ This was brought to light as individuals who had previously had the smallpox vaccine had 85% protection against MPXV.^8^ According to CDC, vaccinia virus vaccines JYNNEOS and ACAM2000 have been used overtime till now to immunize against Mpox.^9^ ACAM2000 vaccine was developed by Acambis, Inc.™ as a single plaque‐purified vaccinia virus derivative of Dryvax which was formerly used for vaccinia virus before its license was withdrawn by CDC in 2008.^10^ The inability of ACAM2000 to control its replication led to the need of a non‐replicating vaccinia virus vaccine in human cells, hence the development of JYNNEOS which is a replication‐deficient smallpox vaccine, for the prevention of smallpox and Mpox viruses.^11^, ^12^

**Figure 1:**
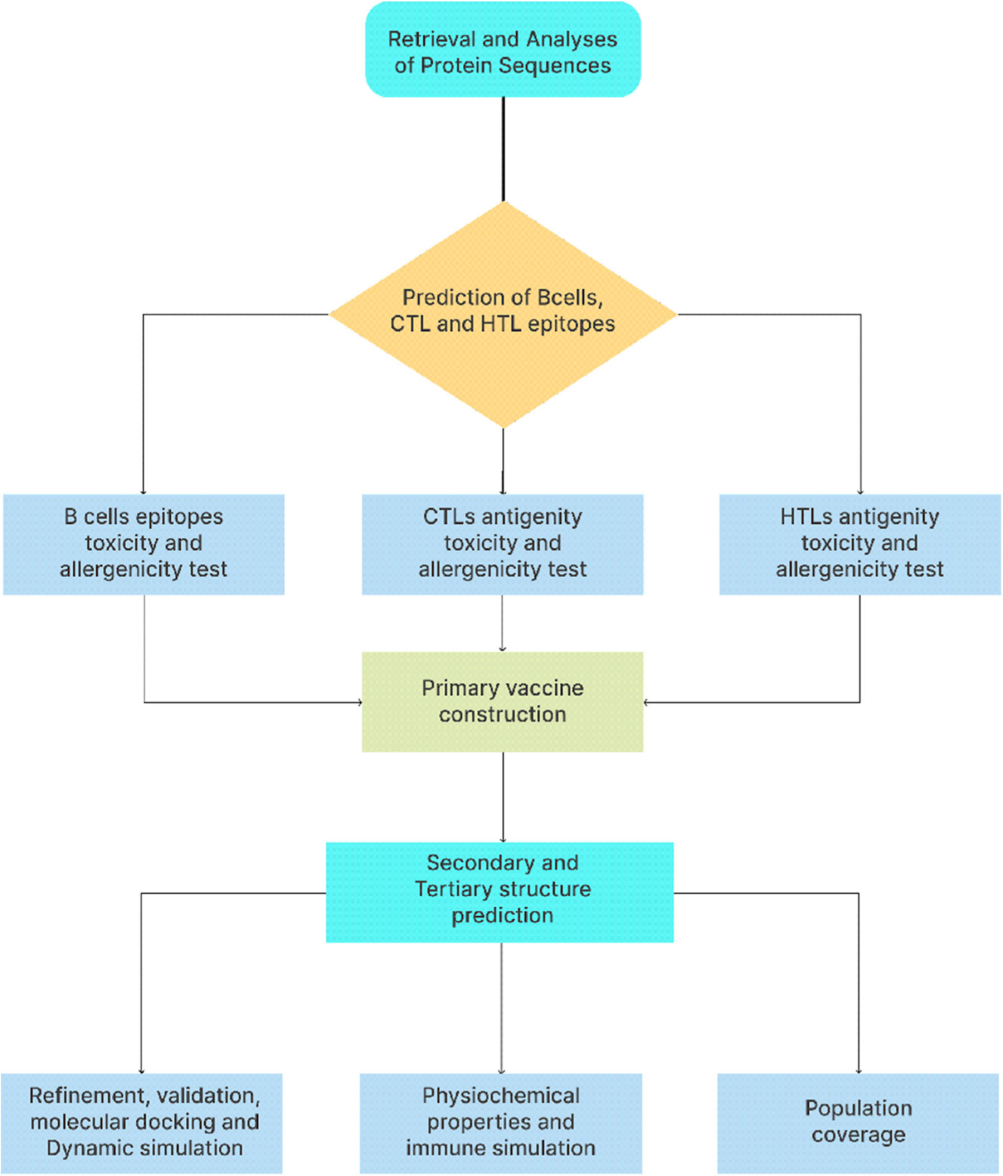
Flowchart representing the overall mRNA vaccine design processes.

In the face of a multi‐country outbreak of MPVX, we have designed an mRNA vaccine for the pox family integrating the vaccinia virus and Mpox virus. The mRNA vaccine stimulates powerful and long‐lasting adaptive immune responses through tumor necrosis factor‐α, alpha Interferon (IFN‐α), and other cytokines secreted by immune cells as a result of the self‐adjuvant capabilities of mRNA vaccine.^13^ (Figure 1 below).

## 2 MATERIALS AND METHODS

### 2.1 Retrieval of target protein sequences

The representative sequences of Mpox and vaccinia used for this study were retrieved from the Virus Pathogen Resource Database (VIPR) (https://www.viprbrc.org/brc/home.spg?decorator=vipr). The sequence data was retrieved across various continents: Oceania, Asia, Africa, Europe, and North America. Sequences on the database from 1990 to 2022 were retrieved. At the end of the collation, a total of 340 Mpox virus and 14 vaccinia sequences were retrieved, respectively, totaling 354 whole‐genome sequences (WGSs). The criteria for the retrieval of sequence data were based on the complete genome, host and date of submission and where humans were chosen as our host.^14^, ^15^

### 2.2 Annotation of retrieved WGS sequences

Three reference antigenic genes of interest—E8L, E7R, and H3L—were downloaded from NCBI and annotated alongside each translated whole‐genome sequence to determine the position of the expression genes in the translated genomes of the pox viruses considered in the study. This annotation process was carried out using MEGA (Molecular Evolutionary Genetic Analysis).^16^

### 2.3 Cytotoxic T lymphocyte prediction

Cytotoxic T lymphocyte (CTL) epitopes were predicted utilizing NetMHCpan–4.1.^17^ The peptide length was restricted to 9 amino acids (9mer) peptides, employing the 12 common allele supertypes available on the server, with a threshold for strong binders set at 0.5% rank and a threshold for weak binders at 2% rank.^14^ The predicted CTL epitopes underwent immunogenicity testing using the IEDB (Immune Epitope Database) server.^18^

### 2.4 Prediction of helper T lymphocytes

The prediction of helper T lymphocyte (HTL) epitope is an important aspect of Immunoinformatics which is used for the development of vaccines. HTL binds to its specific epitope, that is, HTL epitope, which is presented to it in the specialized groove of MHC Class II molecules. HTL epitopes were predicted using the IEDB, MHC‐II binding server, and Human was selected as the host organism. Percentile rank of 0–1.0 was used to determine the candidate epitopes of choice.^19^ Hence, the epitopes with percentile rank lesser than 1 were selected for further downstream analyses. The ability to induce IFN‐γ production, synthesis, and secretion of interleukin‐4 and ‐10, respectively, were also predicted using different Immunoinformatics tools, that is, IFNepitope, IL4PRED,^20^ and IL10PRED, respectively.^21^

### 2.5 Prediction of linear B cell

The identification and prediction of candidate B cell epitopes plays an important role in vaccine design as nature has this to be the region on the surface of a pathogen antigen, where specific antibodies recognize and binds, to trigger immune response.^14^ According to Manavalan et al., to predict the linear B cell epitopes, ABCpred and Bcpred servers were employed.^22^, ^23^

### 2.6 Construction of vaccine's primary structure

The criteria used for the selection of epitopes used for the vaccine construct were based on the antigenicity, non‐allergenicity, and non‐toxicity of our epitopes. There are limitations to the stability and the activity of the mRNA in vivo. There can be an increase to the durability and expression of the structural elements by optimization of the RNA. The 5′ capping was used to protect the mRNA from degrading. Also, UTRs were used for posttranslational gene expression control. HTL, CTL, and LBL epitopes were joined using linkers. The mRNA was constructed using the epitopes that meet the above criteria. LBL and HTL epitopes were linked using (EAAK)_2_. CTL and LBL epitopes were linked using linker AAY which are also used in combination for intra‐CTL epitopes. Adjuvants play a very important role in increasing immunogenicity.^24^

### 2.7 Physiochemical property and secondary structure of the vaccine construct

ProtParam tool was used in predicting the Physiochemical properties such as amino acid composition, theoretical isoelectric point (pI), instability index, in vitro and in vivo half‐life, aliphatic index, and molecular weight of the vaccine construct for both Mpox and vaccinia.^25^, ^26^ The orientation of the protein folding is determined by the protein secondary structure using SOPMA.^27^

### 2.8 Structure modeling, assessment, and validation

The 3D structure of the constructed vaccine was modeled using Phyre 2.^28^ ProSA‐web assessed and validated the protein structure in silico.^29^ The 3D model of the vaccine construct was also assessed for the stereo‐chemical quality using ProCheck bioinformatics tool.^30^

### 2.9 Molecular docking of vaccine against Toll‐like receptors

Molecular docking is a bioinformatics technique adopted for the prediction of the binding affinity between a receptor and its ligand. The possible binding affinity between the mRNA vaccine's construct and human Toll‐like receptor was examined. ClusPro was used to conduct molecular docking between the tertiary structure of the vaccine construct and human Toll‐like receptors (TLR‐2, ‐3, ‐4, and ‐9).^31^

### 2.10 Molecular dynamic simulation

Molecular dynamic simulation is usually employed to determine how a bimolecular system will respond to some changes and to understand the relationships between the sequence structure and function.^32^ In this study, we investigated the interaction between a Mpox vaccine molecule and the TLR which shows the highest binding score using molecular dynamics (MD) simulations that were performed on the Texas Advanced Computing Center (TACC) with Gromacs software. Following established protocols, the vaccine‐receptor complex was solvated with TIP3P water in a box with appropriate ions and minimized with OPLS‐AA force fields. The system was then equilibrated and subjected to a production MD simulation at physiological temperature and pressure using periodic boundary conditions and the leap‐frog integrator. Analysis of the trajectories using Gromacs includes RMSD, RMSF, radius of gyration, and hydrogen bond analysis to elucidate the structural dynamics and interactions between the vaccine and receptor.^33^

### 2.11 Immune response simulation

The C‐ImmSim web server simulated expected immune response features and assessed immunogenicity for all predicted vaccine peptides. Employing a position‐specific score matrix‐based agent‐based computational immune response simulator, C‐ImmSim was used to determine innate and adaptive immune cell and cytokine abundance upon injection of the candidate mRNA vaccine for vaccinia and Mpox.^34^

### 2.12 Population coverage

To determine the population covered by the mRNA vaccine construct, MHC I and II binding alleles were predicted using the IEDB MHC I and II prediction servers, respectively.^35^ The predicted alleles and their respective epitopes were used to determine the population coverage across the globe using the IEDB population coverage prediction tool. The algorithm of this tool was designed to calculate the HLA genotypic frequency and percentage of individuals expected to respond to the given vaccine.^36^

## 3 RESULTS

### 3.1 Prediction and evaluation of CTL, HTL, and B cell epitopes

Mpox viral WGSs were exclusively obtained from five continents: Asia, North America, Europe, Africa, and Oceania, comprising 2, 28, 106, 39, and 1 sequences, respectively. Additionally, 14 sequences of vaccinia virus were retrieved. Predictions yielded 60 CTL epitopes for Mpox virus and 213 for vaccinia virus, while 60 and 133 HTL epitopes were projected for Mpox and vaccinia viruses, respectively. B‐cell epitope predictions resulted in 99 for Mpox virus and 72 for vaccinia virus. Rigorous analysis involving immunogenicity, toxicity, and interferon production for the epitopes identified one HTL epitope, six CTL epitopes, and five LBL epitopes meeting the criteria, forming the basis for mRNA vaccine construction.

### 3.2 Designing, primary and secondary structural evaluation of mRNA vaccine

**Figure 2:**
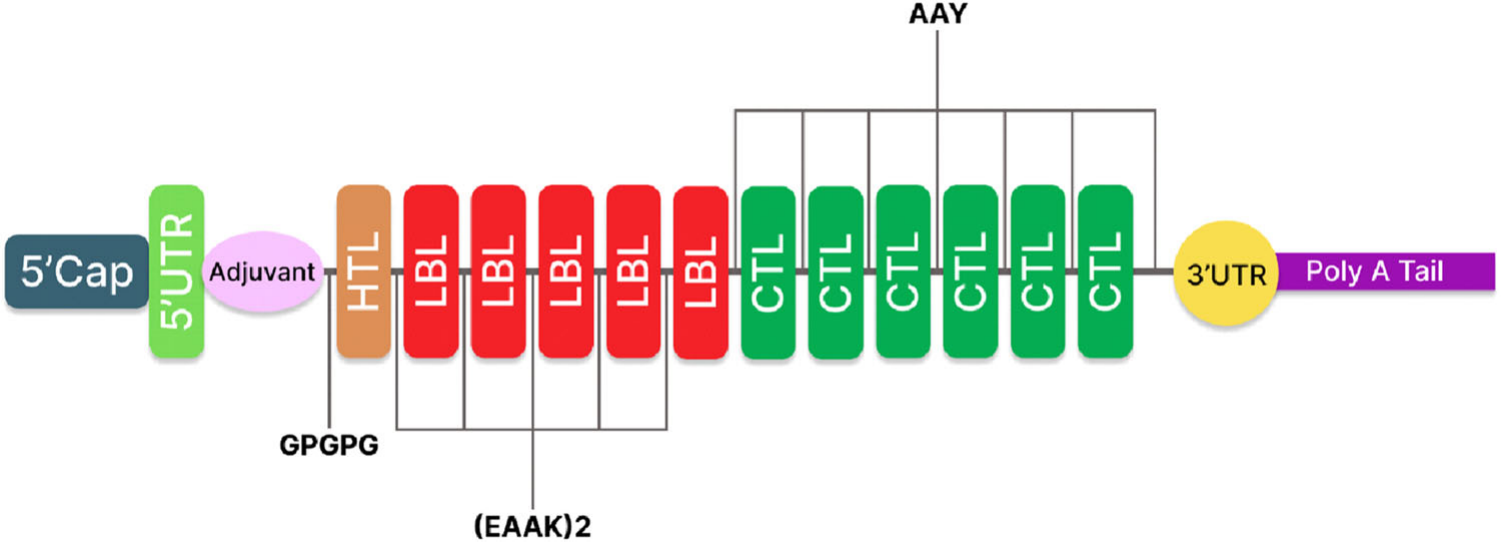
Schematic representation of the mRNA final vaccine construct.

In designing the vaccine's primary construct, the epitopes that passed the evaluations were combined with some co‐translational residues following the order of Oluwagbemi et al. and Oladipo et al.^14^, ^15^ The 5′cap, 5′UTR, KOZAK sequence, and signal peptide were combined together with the adjuvant (Beta‐defensin‐1) and then linked to HTL epitope with the help of linker (GPGPG). Also, (EAAK)_2_ link (HTL to LBL) and (AAY) link (LBL to CTL), respectively as shown in Figure 2 below.

**Figure 3:**
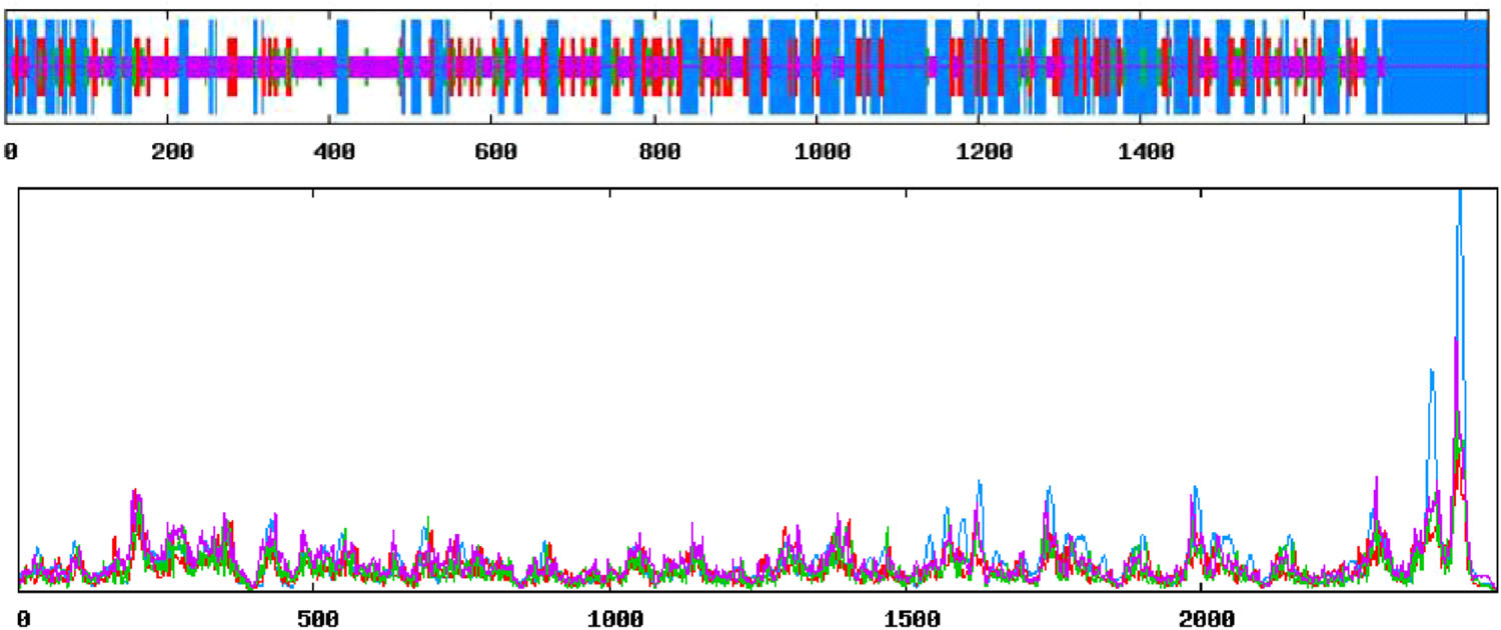
Secondary structure of the mRNA vaccine construct.

After the construction, the physicochemical properties were evaluated which revealed the molecular weight of the vaccine to be 198 kDa. The computed theoretical pi was 6.97 while the GRAVY score is −0.163 which indicates a hydrophilic nature of the vaccine. Aliphatic index score (77.84) indicated that they are thermally stable as well as they contain high amounts of hydrophobic amino acid. The vaccines are probably unstable as a result of their instability index (46.14), which is greater than 40 with an estimated half‐life of 30 h in mammalian reticulocytes (in vitro), >20 h in yeast (in vivo) and >10 h in *Escherichia coli* (in vivo). Results from the secondary structure show that the vaccine consists of 42.45% Alpha Helix, 16.14% Extended Strand, 7.39% Beta turn, and 34.03% Random coil as depicted in Figure 3 below.

### 3.3 Population coverage of the vaccine construct

**Figure 4:**
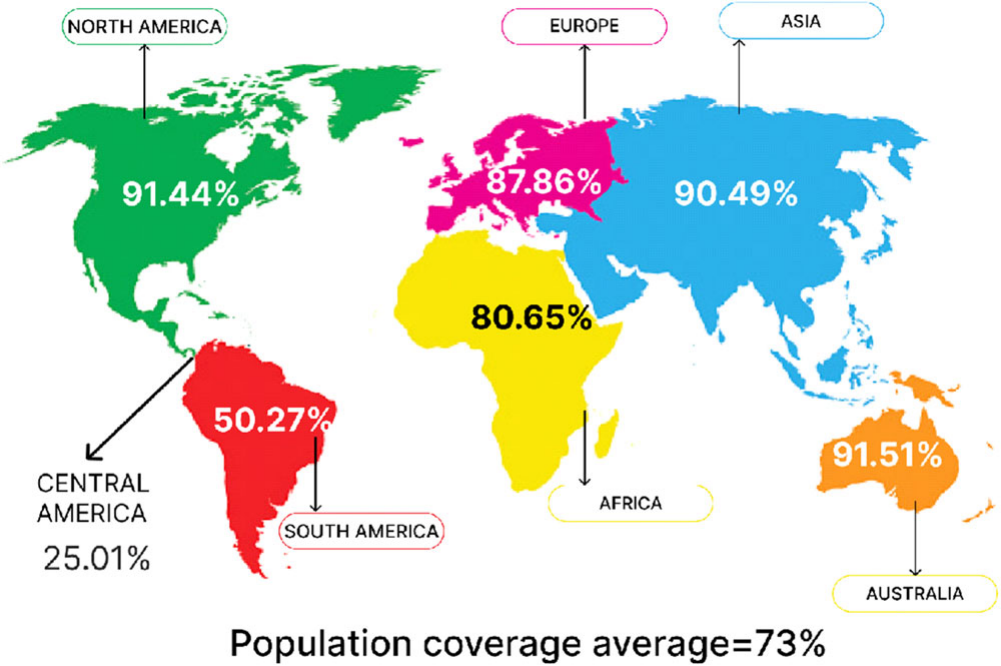
Predicted population coverage of the mRNA vaccine construct.

The combined coverage for the population coverage predicted the mRNA vaccine with the capability to induce immune response in 90% of the world's population. Europe had the highest population coverage for combined MHC Class I and Class II epitopes (99.86%), followed by North America (99.59), East Asia (98.6%), Northeast Asia (96.09%), South Asia (97.6%), Southeast Asia 95.85%), Southwest Asia (94.31%), Central Africa (89.83%), Central African Republic (71.34), East Africa (92.82), North Africa (98.1%), South Africa (94.81%), South Africa Black (94.01%), South Africa other (97.61%), and West Africa (96.5%) as shown in Figure 4 below.

### 3.4 Tertiary structure of the vaccine construct

**Figure 5:**
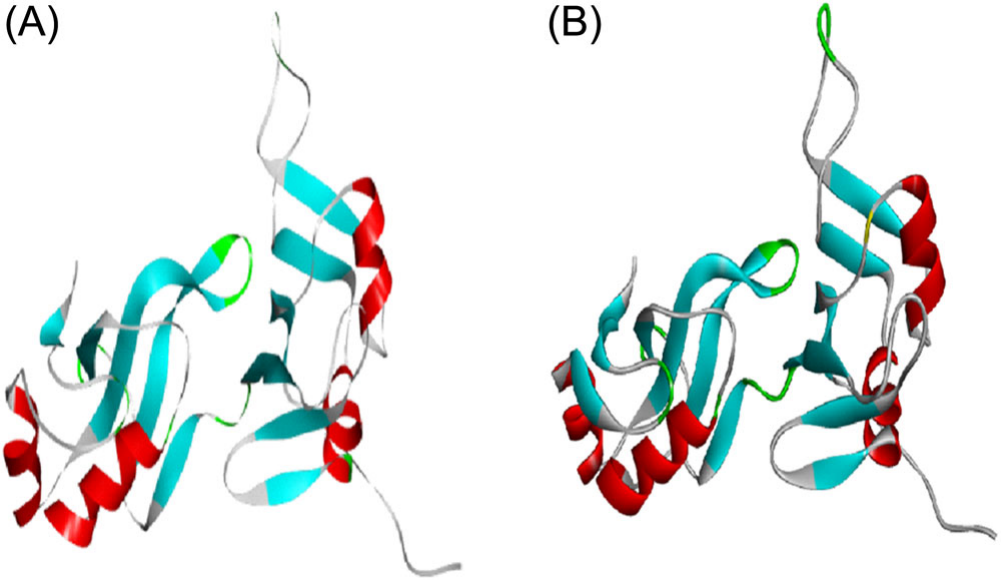
(A) Predicted tertiary structure of Mpox mRNA vaccine construct. (B) Tertiary structure refinement of Mpox mRNA vaccine construct.

**Figure 6:**
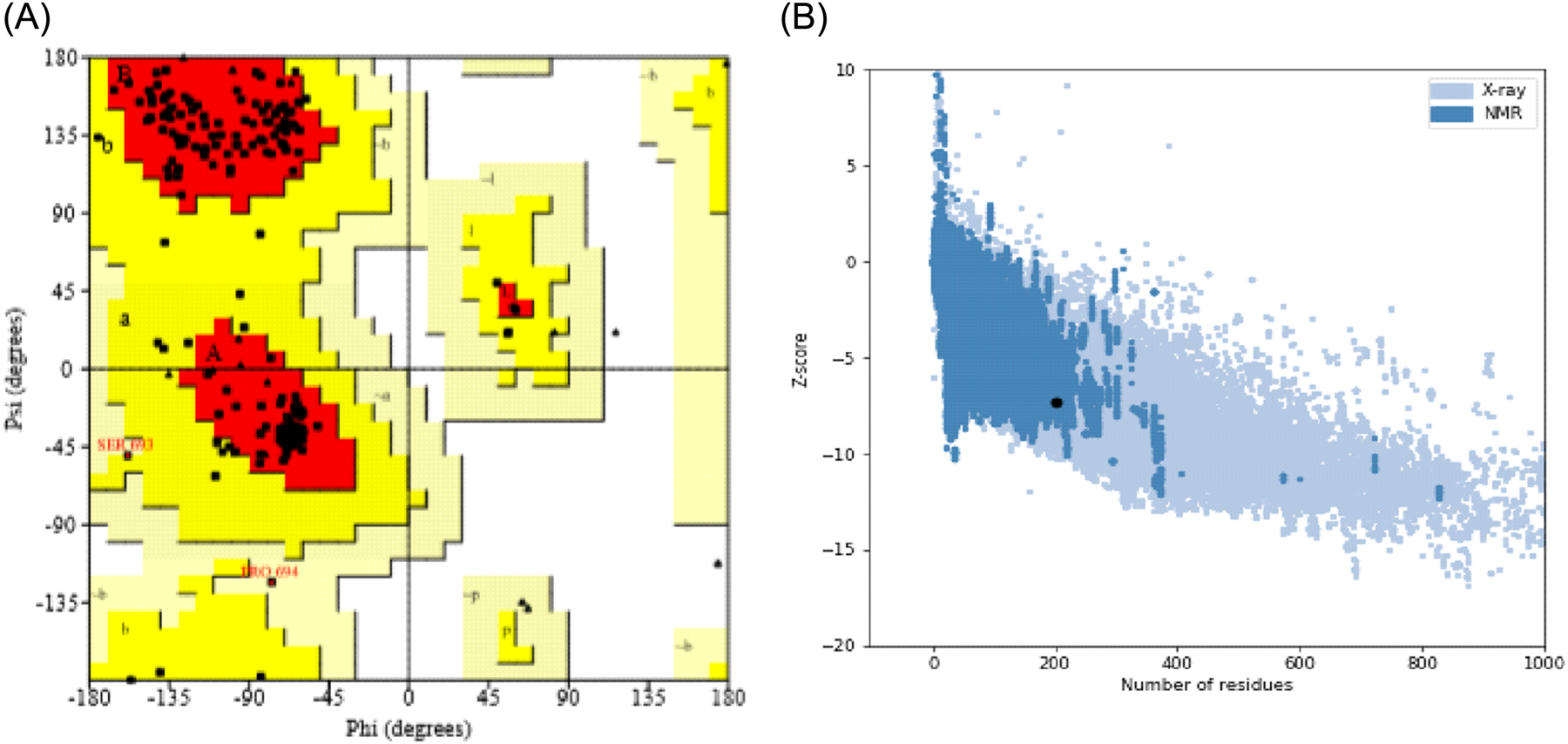
(A and B) Ramachandran plot and *z*‐score graph representing tertiary structure validation.

The tertiary structures of the constructed mRNA vaccine were generated using PHYRE2 server and ProSA‐web was used for refinement of the 3‐D structure generated from PHYRE2 server as shown in Figure 5A,B below. The final 3D model structure of the vaccine construct and Ramachandran plots are depicted in Figure 6A,B below. The obtained models were then refined through several structure perturbations and subsequent structural relaxation using Galaxyweb server. Ramachandran plot analysis showed 88.8% residues in favored regions (A, B, and L), 10.7% residues in additional allowed regions (a, b, l, and p) and 0.6% residues in generously allowed regions (~a, ~b, ~l, and ~p). This analysis shows the reliability and stability of the predicted structures.

### 3.5 Molecular docking of the vaccine construct and TLR complex

For an effective and stable immune response, the interaction between the immune cell and the vaccine is required. The docking of all vaccine constructs was performed with TLR‐2, TLR‐3, TLR‐4, and TLR‐9, as diagrammatically shown in Supporting Information S1: Figure 1. The ClusPro program produces 18 different clusters, with high interaction energies. The top five clusters had energies of −646.6, −730.6, −771.8, −739.1, and −704.6, respectively. After analyzing all vaccine constructs, the first cluster was selected of the TLR‐3 was selected from other TLRs based on the energy score of which it had better energy than the other Toll‐like receptors.

### 3.6 MD simulation

**Figure 7:**
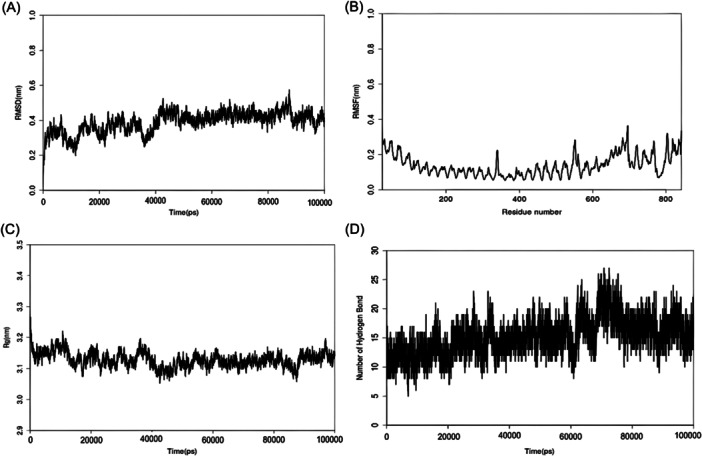
Molecular dynamics simulation for the vaccine construct. (A) The root mean square deviation. (B) Root mean square fluctuations. (C) Radius of gyration. (D) Number of Hydrogen atoms interactions in the simulation.

We investigated the interaction between a Mpox‐vaccine molecule and its TLR‐3 (The Toll‐like receptor with the highest binding score), using 100 ns MD simulations performed on TACC with Gromacs software. Analysis of the trajectories using Gromacs revealed insights into the interaction: the relatively stable RMSD value between 0.2 and 0.4 nm over a period of simulations suggests vaccine stability, RMSF shows there is a constant and potential interaction site, a relatively constant Rg indicates a compact vaccine conformation, and a significant number of hydrogen bonds indicate strong binding affinity. These results provide a deeper understanding of the structural dynamics and potential binding mechanisms between the Mpox vaccine and TLR‐3. Figure 7A–D below shows there is absolutely an interaction between amino acid residues of the mRNA vaccine constructs and the Toll‐like Receptor.

### 3.7 Immune response simulation

The immune simulation investigation aimed to scrutinize immune interactions and assess the potential induction of adaptive immunity in vaccine complexes. Utilizing the C‐IMMSIN server, the effectiveness of vaccine dosage per administration on the immune system was studied. The results indicated a significant increase in the primary immune response after each injection dose, with observable gradual fluctuations in immunoglobulin levels. Additionally, the secondary immune response exhibited an upward trend. Notably, increased rates were observed in active B cells, plasma B cells, helper T cells, and regulatory and cytotoxic T cells. The findings revealed that both primary and secondary immune responses were mediated through the T cell population (helper T cells and cytotoxic T cells) and sustainable memory cells, underscoring the efficacy of our designed mRNA vaccine candidate. In silico immune simulation was employed to analyze the vaccine construct's ability to elicit an immune response. The immune responses from the mRNA vaccine construct simulation are illustrated in Supporting Information S1: Figure 2.

## 4 DISCUSSION

Vaccines are legendary for their prevention of various diseases and illnesses. Diseases like Smallpox, which has been eradicated polio, measles, and other diseases have been reduced drastically around the world through vaccines.^37^ Vaccine approaches all this while has solely been dependent on conventional means which includes live attenuated, inactivated pathogens and subunit vaccines, but in the cases of infective pathogens that can evade the adaptive immune response, vaccine development has been a serious challenge.^38^, ^39^ Instances like Mpox virus have led researchers to look towards the mRNA vaccine as therapeutics for combating diseases that can evade the adaptive immune response.^40^ As a result of the noninfectious and non‐integrating feature of mRNA vaccine,^41^ it has been chosen to be more preferred over other types of vaccines like the DNA vaccine, subunit, live attenuated and killed, which has made it more beneficial and effective.^42^ All these advantages of mRNA vaccine coupled with the fact that it possesses no insertional mutagenesis risk and does not have any potential to cause infection as it is being normally degraded by cellular processes^38^, ^43^ has raised the call to our study to design an mRNA vaccine for the re‐emerging Mpox virus which will also be effective against vaccinia virus.^31^

Three expressed genes, including the H3L gene, E8L gene, and E7R genes, were predicted by Vaxijen server to be antigenic in both the Mpox and vaccinia viruses.^44^ Considering the fact that Mpox and small viruses are both pox viruses, the vaccine was designed to cater to both. Their antigenic proteins were explored for our vaccine candidate. The genes were predicted from a list of WGSs downloaded from a database that is specific to viruses known as VIPR.^45^

The vaccine, which was constructed from the 11 epitopes (B cell, CTL, and HTL) predicted from the antigenic genes of the isolates, was combined with the linkers EAAKEAAK and AAY linkers. The adjuvant β defensin‐1 was adopted for the construct because it boosts vaccine efficacy by increasing the vaccine's ability to induce immune responses^46^; The adjuvant was then connected to the already linked epitopes with the GPGPG linkers. According to Petel and LeSage's study in 2014, *Linkers* have been observed to play a crucial role in the assistance of the vaccine to produce a significant amount of antibodies and also to act as an independent immunogen.^47^

The global populations to be covered are to be put into consideration, being a function of the difference in the HLA allele across the globe, geographical regions and ethnic groups.^48^ The selected T cell epitopes exhibited a higher individual percentage cover when queried with the entire world population. The HLA hits across the entire population revealed that approximately 68.8% of the world's individuals are capable of responding to six CTL epitopes and 69.46% of individuals for HTL epitopes.

To prevent cross‐reactivity whenever the vaccine is introduced into the body. The epitopes obtained were blasted against the genes expressed by humans, and the result which came out in favor of our construct, satisfies one of the requirements that validate the safety of the vaccine for use as therapeutic and not causing inflammation in the long run.

To define the efficacy and the effectiveness of an mRNA vaccine, the knowledge of the physicochemical properties of the vaccine is needed.^48^ The constructed vaccine has a molecular weight of 198 kDa and theoretical pI of 6.90. This molecular weight is higher than the value considered good for the vaccine because proteins having lower molecular weight proves easy in purifying them and can efficiently be subjected to vaccine development.^49^ The predicted theoretical pI depicts that the vaccine is slightly acidic. The instability index of 46.14 computed shows that the vaccine is slightly unstable which is a characteristic of mRNA vaccines, which gives us conscious information of the environment needed to maintain or preserve the mRNA vaccine from getting denatured. Also, 30‐h half‐life estimated for Mammalia reticulocyte in vitro depicts the time it takes for the protein to disappear inside the cell after synthesis.^50^ The aliphatic index of a protein is the relative volume of protein occupied by aliphatic side chains which indicates the stability of the protein against heat. The vaccine has a value of 77.84 as the aliphatic index which indicates a relatively high thermal‐stability. The −0.163 GRAVY index of the constructed vaccine depicts the hydrophobic nature of the designed vaccine.^50^ The binding affinity between a receptor and its ligand can be defined by the energy released during spontaneous bond formation between the two and the lower the energy, the more tightly bound the receptor is to its ligand. The results of the docking of TLR‐2, ‐3, and ‐9 with the vaccine construct show from the score that TLR‐3 has the highest binding affinity to the vaccine which implies that the vaccine antigen will likely bind to TLR‐3 for activation of innate immune cells as reported by Tahir et al.^51^ In addition, the understanding of biological regulation and its mechanisms provides a conceptual and theoretical knowledge for the discovery and design of drug and vaccine targets. Hence, the study of the interaction between protein and ligand is essential.

The result of the dynamics simulation from the RMSD plot shows the Vaccine‐TLR complex been relatively steady binding which is also an advantage to the vaccine's immunogenicity. Furthermore, based on the Ramachandran plots and *z*‐score which was used to determine the quality of the vaccine's 3D model. 88.8% of the amino acid residues are in the favored region while 10.7% are in the allowed region which does not really contribute to the goodness of the vaccine. The description of how the vaccine's 3D model compares with experimentally determined protein structures of similar sizes from different sources from the *z*‐score shows that the model's zone in the *z*‐score plot is acceptable.^29^ The vaccine is also able to improve immune response against antigens in subsequent infections and also the display of increased B cell and T memory cell activities within the first 5 days and remained constant for weeks. It also displayed increased titer of the immunoglobulin antibodies within the first 20 days as shown from.^52^ The entire results favor the candidacy of the mRNA vaccine when compared with other studies from computational standpoint,^5^, ^53^, ^54^ and therefore further analyses via in vivo and in vitro techniques need to be performed to validate this claim.

### 4.1 Limitations

While the computational design of the mRNA vaccine against vaccinia and Mpox viruses shows promising results from the computational approach, we acknowledged several limitations to this candidate's vaccine.^55^ First, the in silico predictions and simulations, although very robust, but cannot fully replicate the complexity of biological systems. Therefore, the actual immunogenicity and safety of the vaccine need to be validated through extensive in vitro and in vivo studies. Second, the study's reliance on available genomic data may not account for all possible viral mutations, potentially affecting the vaccine's efficacy against emerging strains. Additionally, the predicted population coverage, while high, may not fully represent the genetic diversity of global populations, necessitating further validation in diverse demographic groups. The stability and delivery mechanisms of the mRNA vaccine also require optimization to ensure effective administration and sustained immune response. Finally, the potential for unforeseen adverse effects, particularly in immunocompromised individuals, underscores the need for comprehensive clinical trials to establish the vaccine's safety profile. Addressing these limitations through rigorous experimental validation and clinical testing is crucial for the successful development and deployment of this candidate mRNA vaccine.

## 5 CONCLUSION

The development of a safe and accessible form of the vaccine is crucial, especially in the context of combating Mpox and vaccinia viruses. The global prevalence of Mpox is currently a significant concern, with no approved efficacy vaccine or therapy. Hence, the development of mRNA vaccines becomes imperative to address this health challenge. In this study, computational methods were employed to design an mRNA vaccine, and the comprehensive results indicate the constructed mRNA vaccine as a promising and efficient candidate. Further validation through in vivo and in vitro techniques is essential to substantiate this assertion.

## AUTHOR CONTRIBUTIONS

Elijah K. Oladipo and Helen Onyeaka: Conceptualization, experimental design, supervising. Daniel A. Adediran, Olanrewaju D. Oyelakin, Abdulsamad O. Aiyelabegan, Elizabeth O. Olajide, Victoria O. Olatayo, Kaothar P. Owolabi, Boluwatife A. Irewolede, and Esther M. Jimah: Data retrieval, analysis and wrote the first draft of the manuscript. Daniel A. Adediran, Seun E. Olufemi, Yewande B. Shittu, Rhoda O. Olugbodi, Hezekiah A. Ajala, Raji A. Rukayat, and Deborah O. Olayiwola: Data analysis and result interpretation. Julius K. Oloke, Daniel A. Adediran, Seun E. Olufemi, Taiwo O. Ojo, Boluwatife A. Irewolede, Olumide F. Ajani, Olatunji M. Kolawole, Olumuyiwa E. Ariyo, and Helen Onyeaka: Data curation, critical review, and critical editing. All authors agreed to the final version of the manuscript.

## CONFLICT OF INTEREST STATEMENT

The authors declare no conflict of interest.

## Supporting information

Supporting information.

## Data Availability

The data that support the findings of this study are available from the corresponding author upon reasonable request.
