# Supplementary material for: Exploring computational approaches to design mRNA Vaccine against vaccinia and Mpox viruses
Source: Immun Inflamm Dis. 2024 Aug 16;12(8):e1360. doi: 10.1002/iid3.1360 (PMC11328121; doi:10.1002/iid3.1360)
Supplement: Supplementary file 1 — Supporting information. [file IID3-12-e1360-s001.docx]

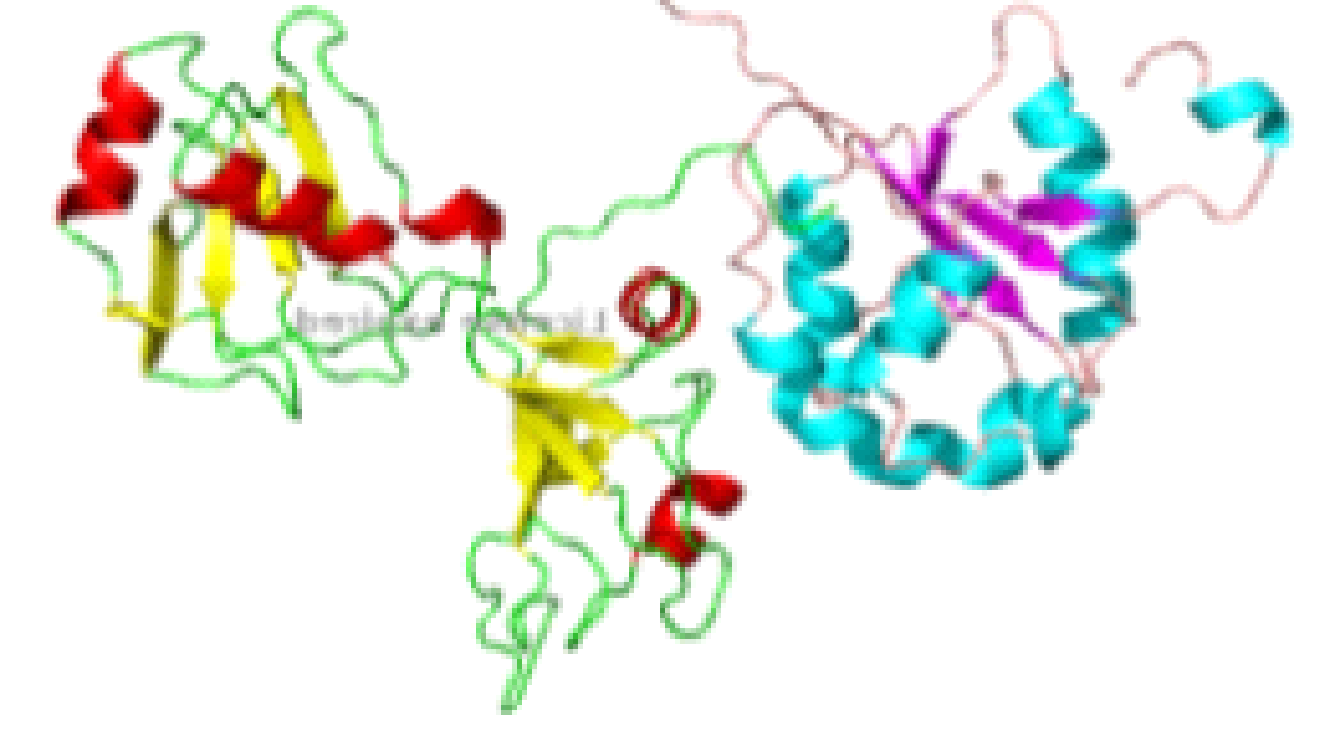

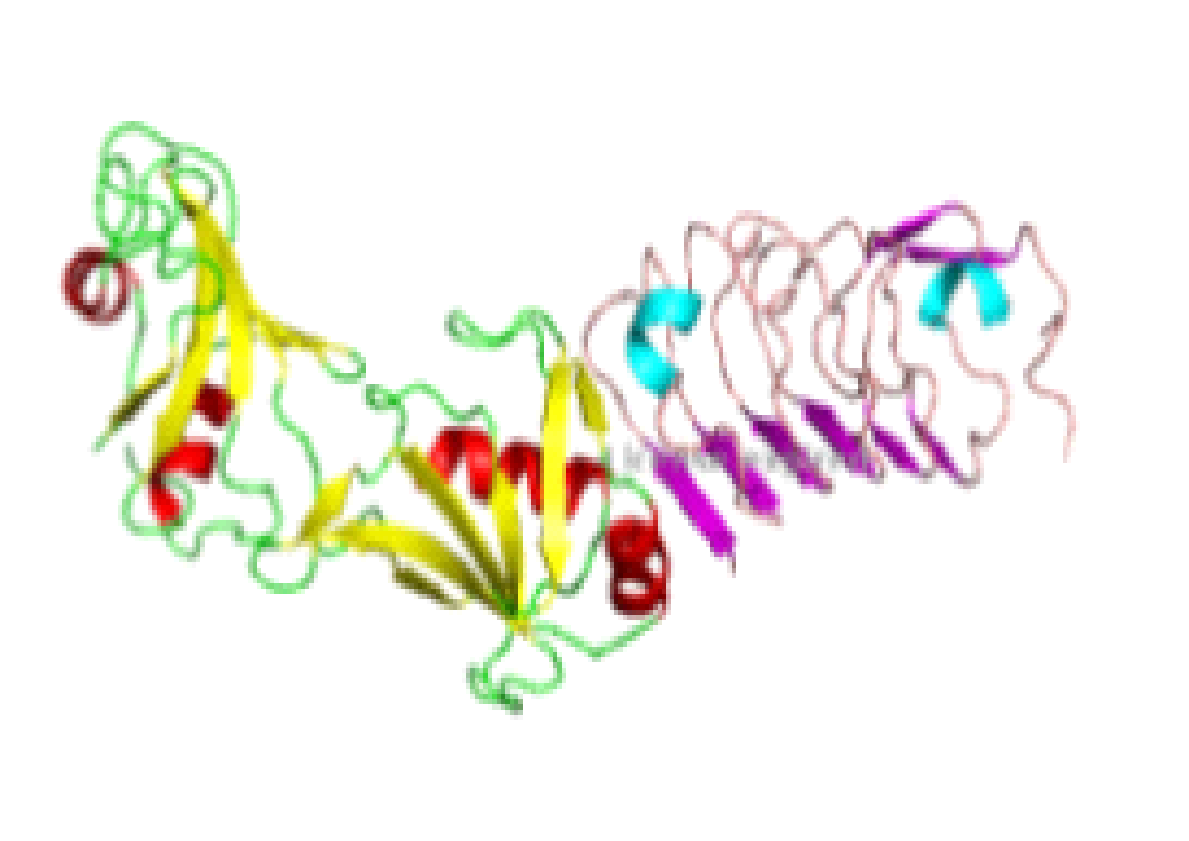


1a 1b
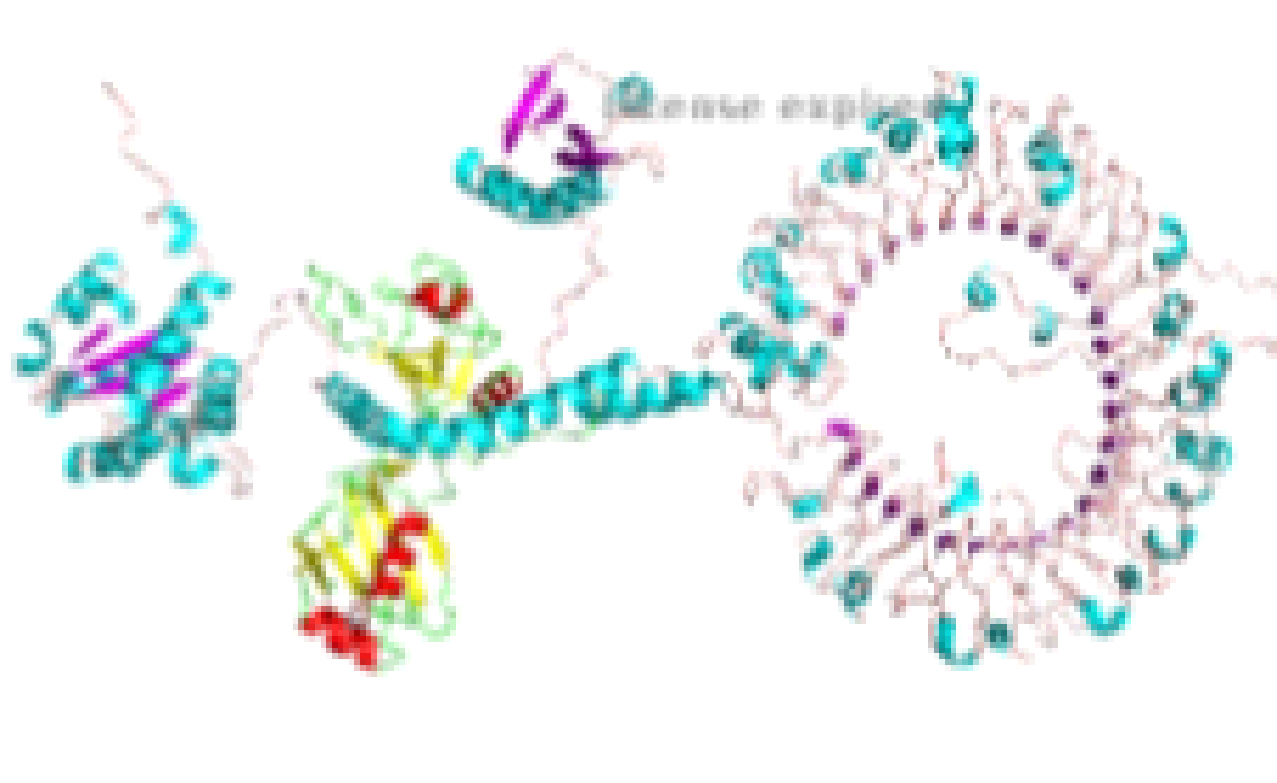

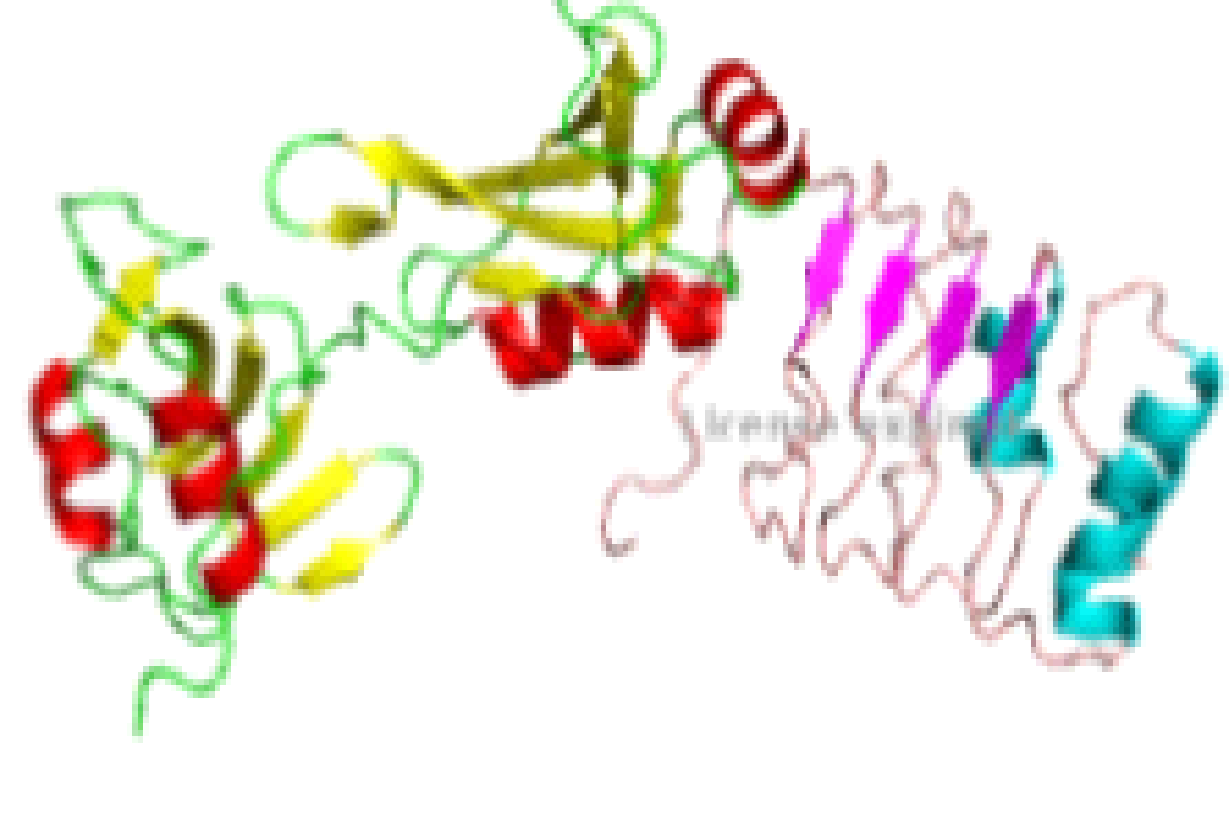


1c 1d

Figure 1a, 1b, 1c and 1d: Molecular Docking of the construct with TLR:TLR 2, TLR 3, TLR 4 and TLR 2 respectively.

Figure 2.0: **Antigens and immunoglobulins**. Antibodies are subdivided by isotypes
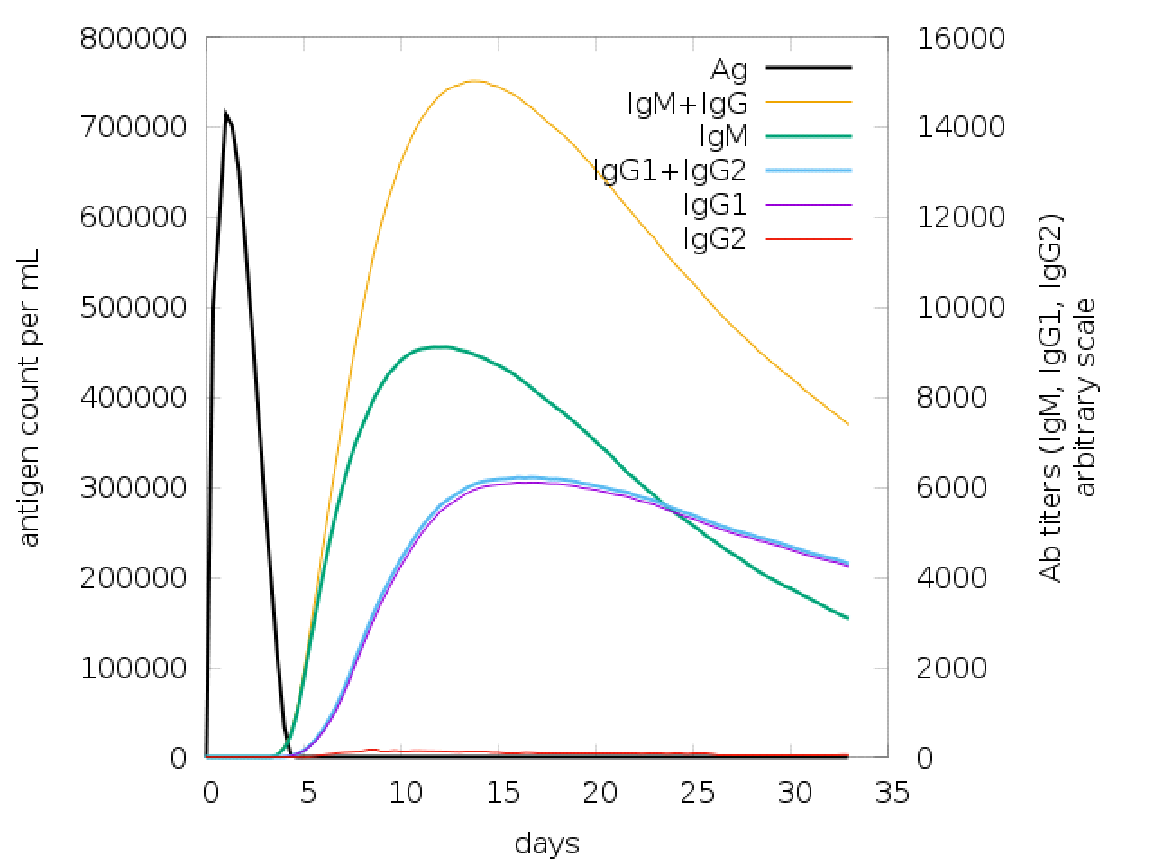


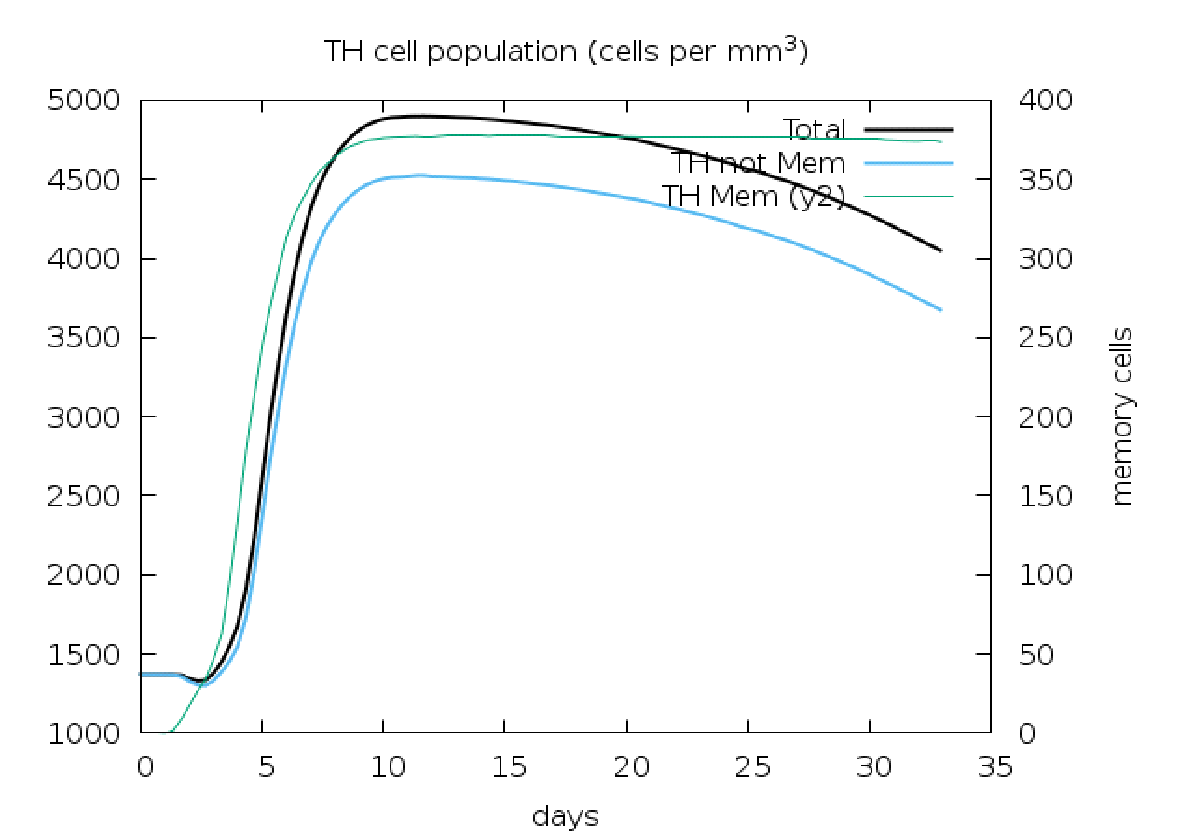


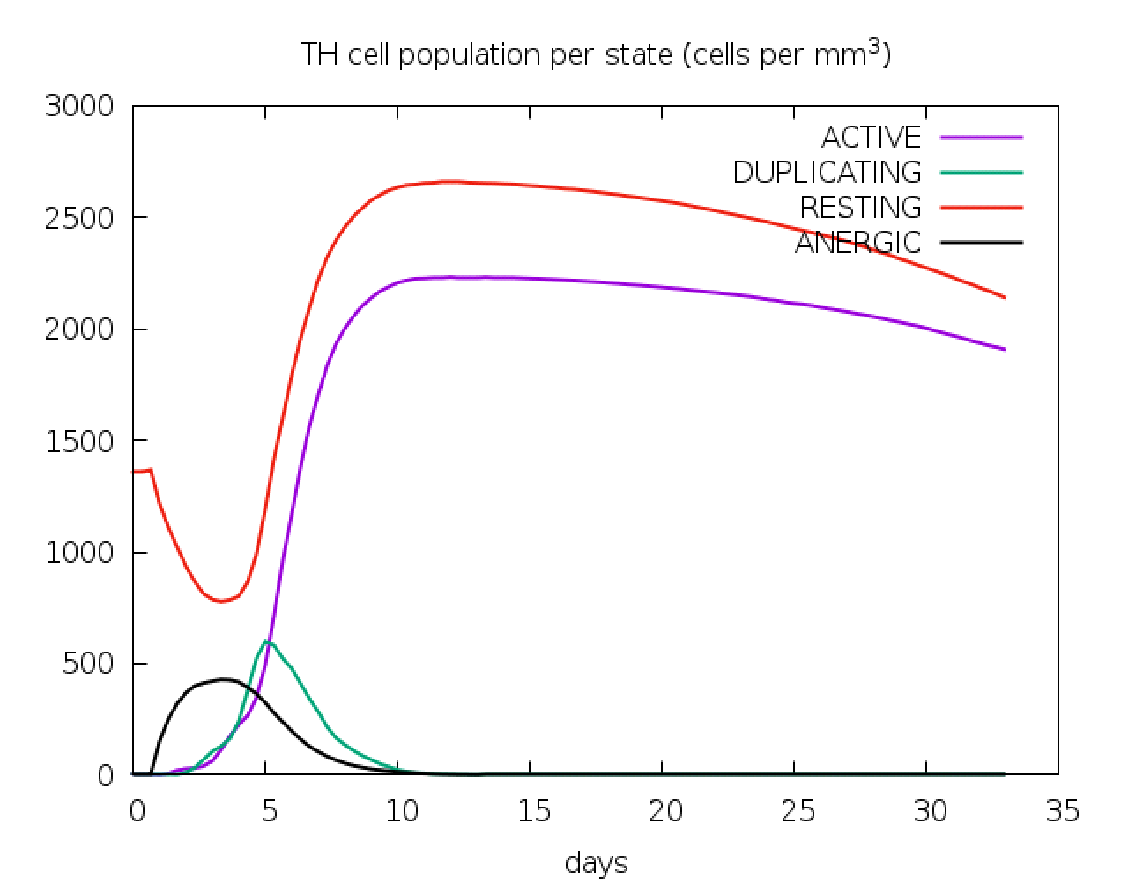


A B

2.1: (A) CD4 T-helper lymphocytes count. The plot shows total and memory counts (B) CD4 T-helper lymphocytes count subdivided per entity-state (i.e. active, resting, anergic and duplicating)


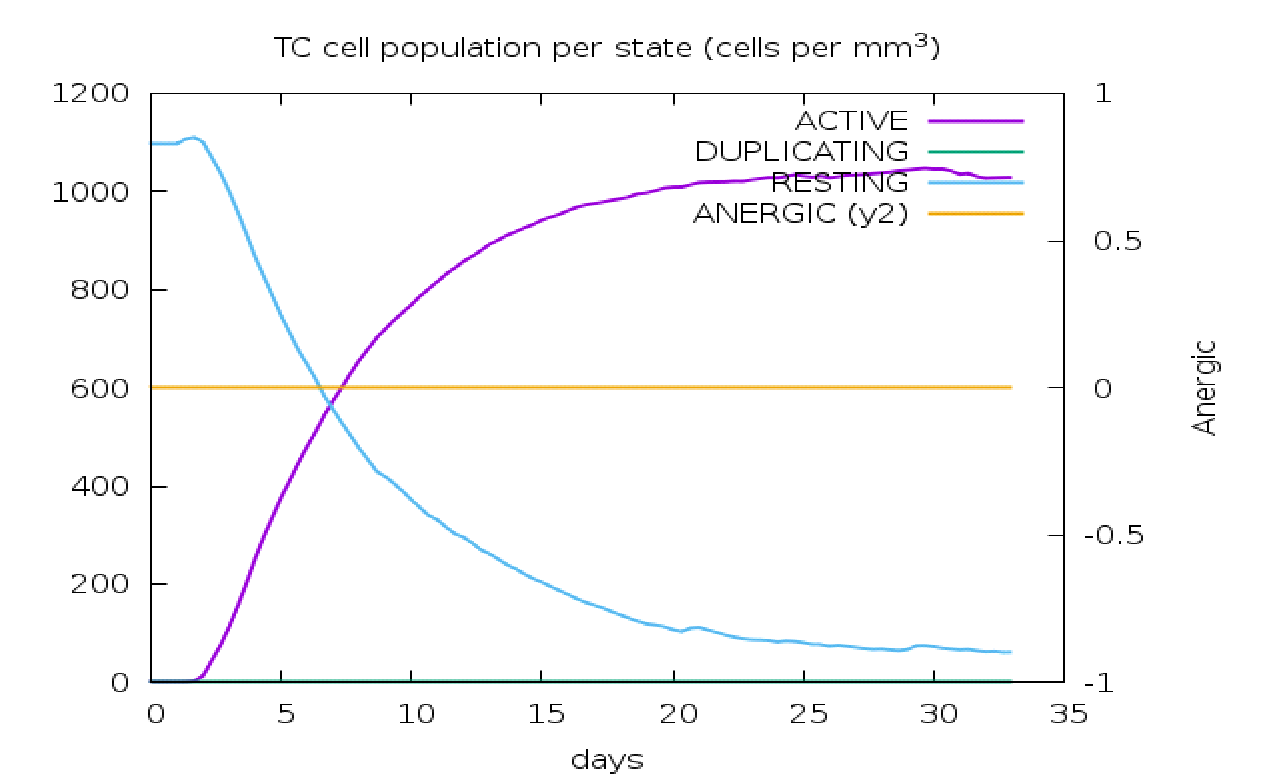

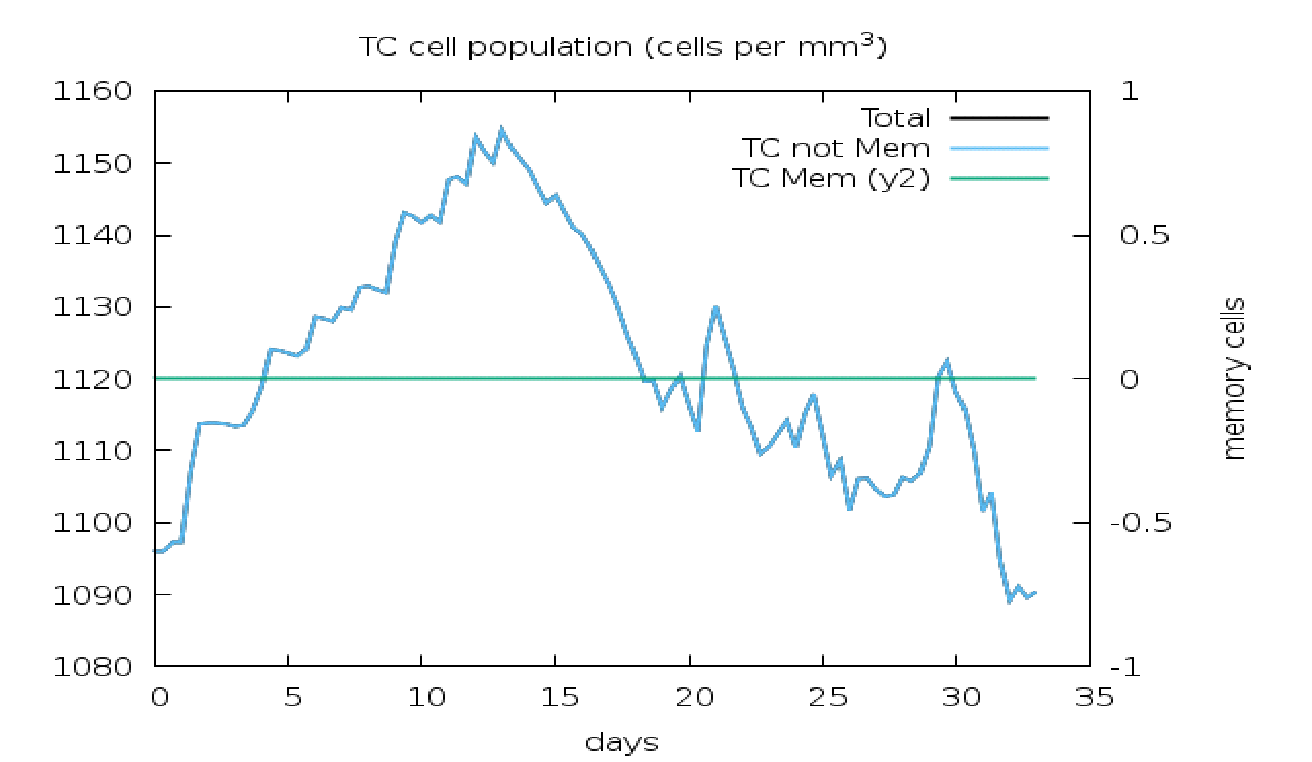


A B

2.2: (A)CD8 T-Cytotoxic lymphocytes count. Total and memory shown (B) CD8 T-Cytotoxic lymphocytes count per entity-state


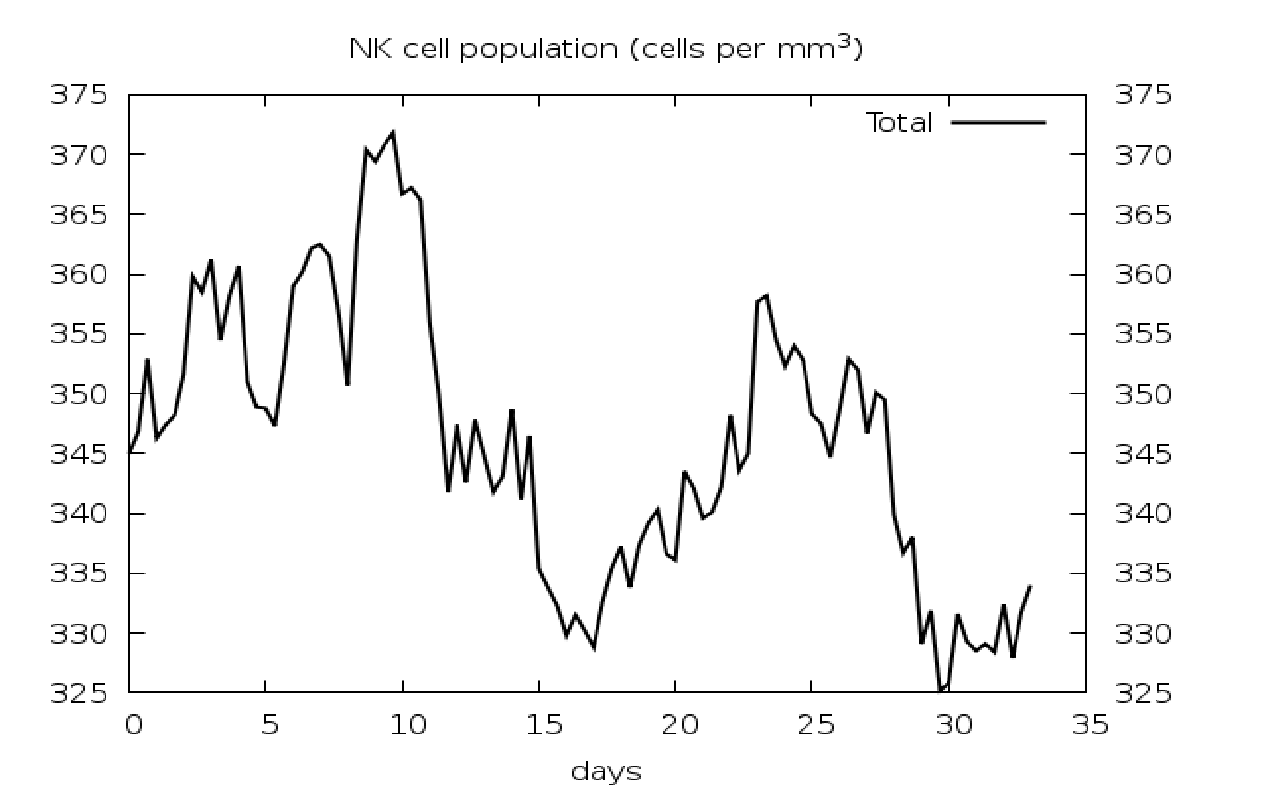

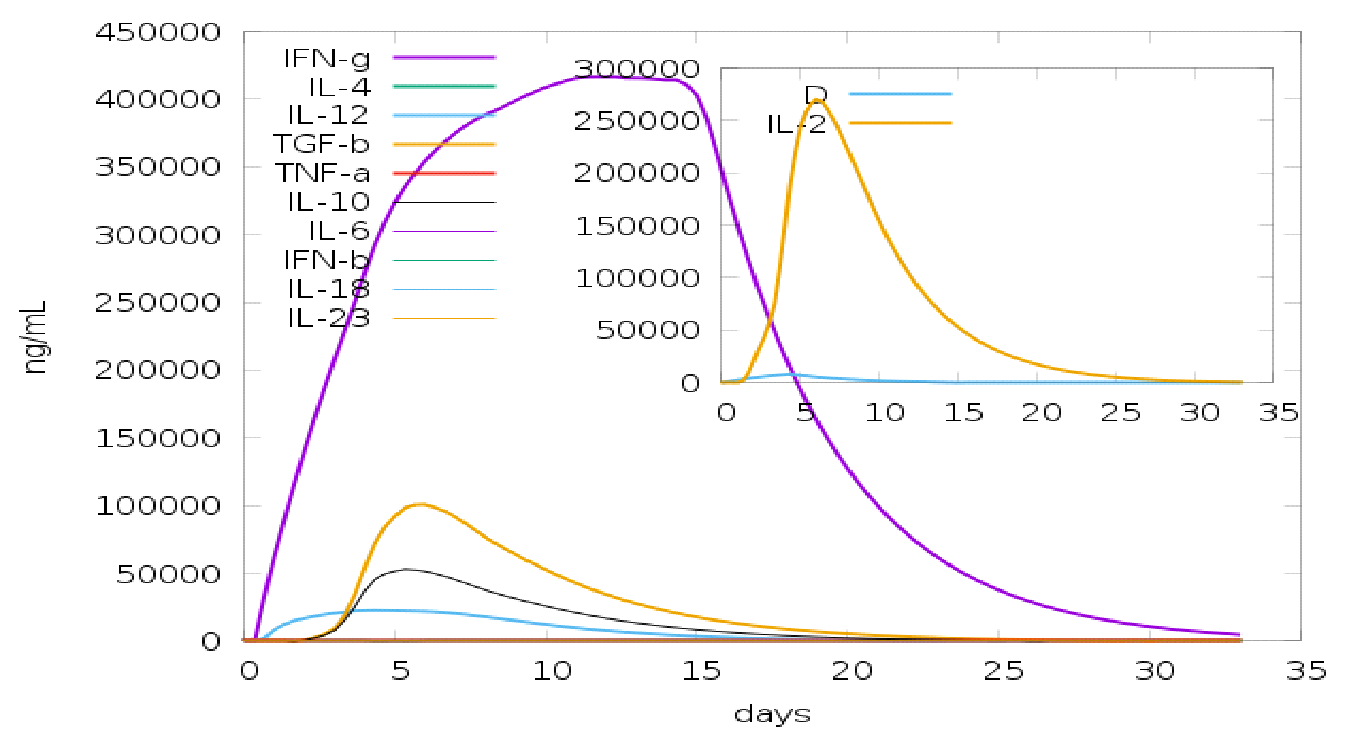


A B

2.3: (A) Natural killer cells Total count (B) Cytokines. Concentration of Cytokines and interleukins. D in the inset plot is danger signal


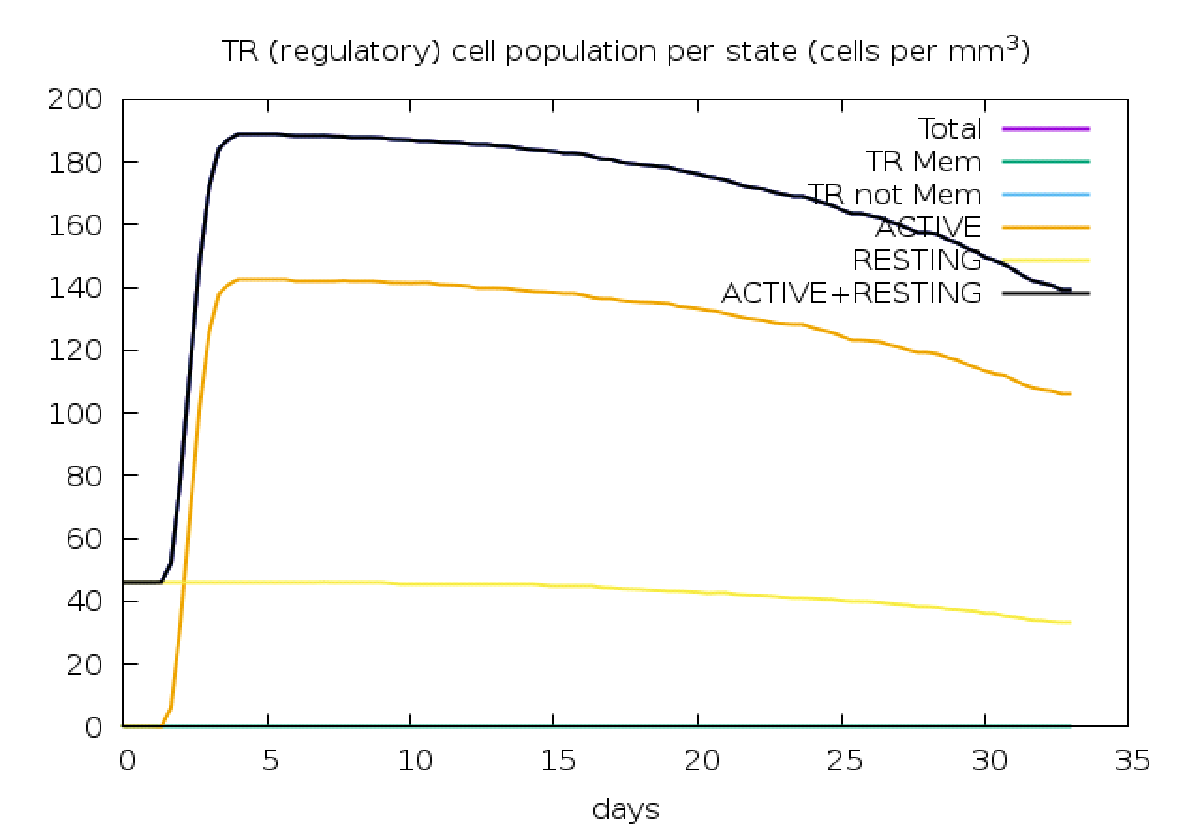

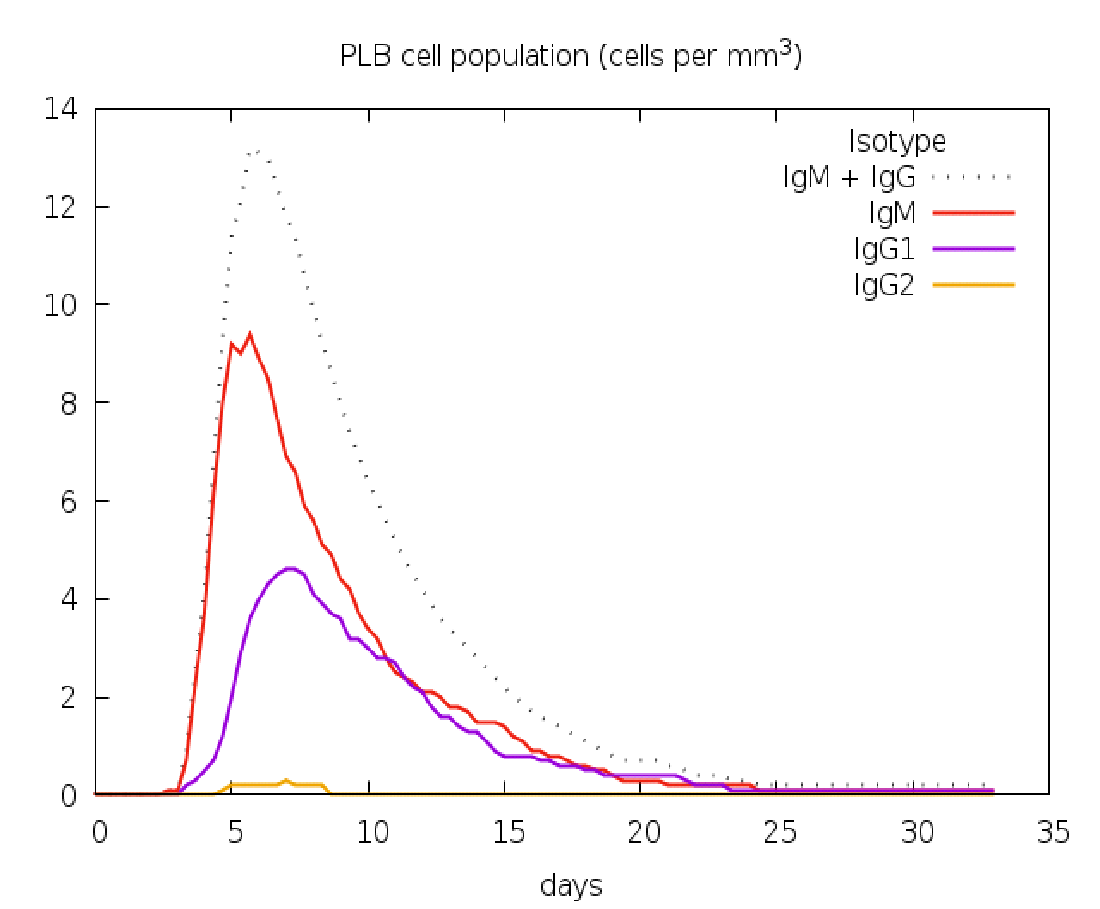


A B

2.4: The PLB population (cells per mm^3^) (B) The relutatory T cells population per state ( cells per mm^3^)


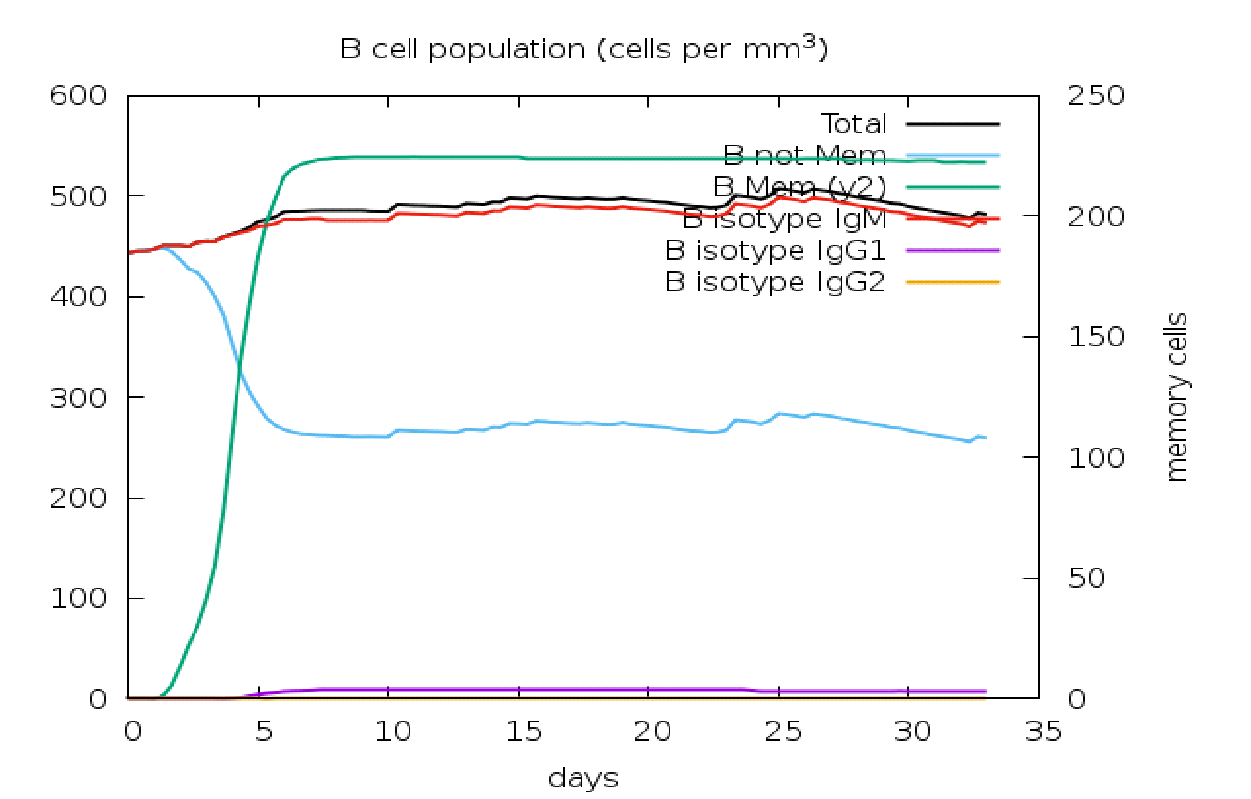

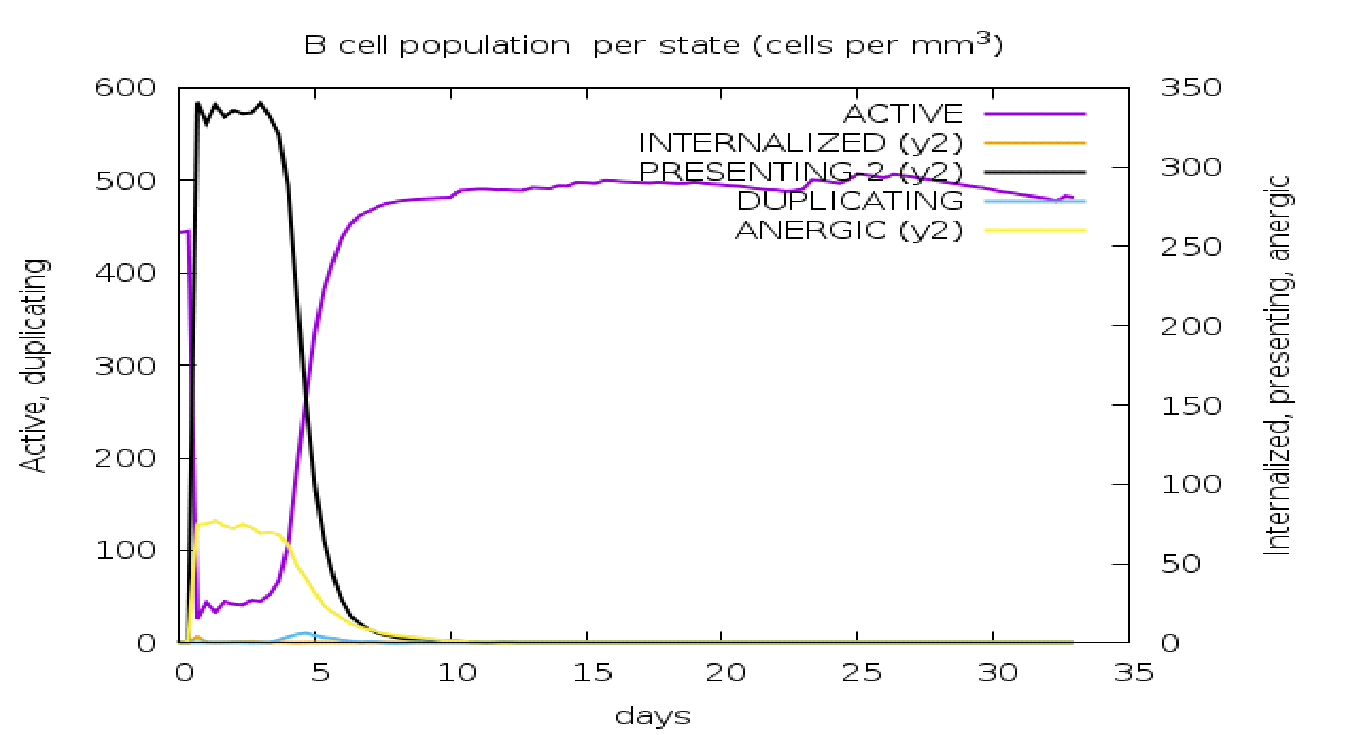


A B

2.5: (A) B lymphocytes: total count, memory cells, and subdivided in isotypes IgM, IgG1 and (B) IgG2 and the B-lymphocytes population per entity-state (i.e. showing counts for active, presenting on class-II, internalised the Ag, duplicating and anergic


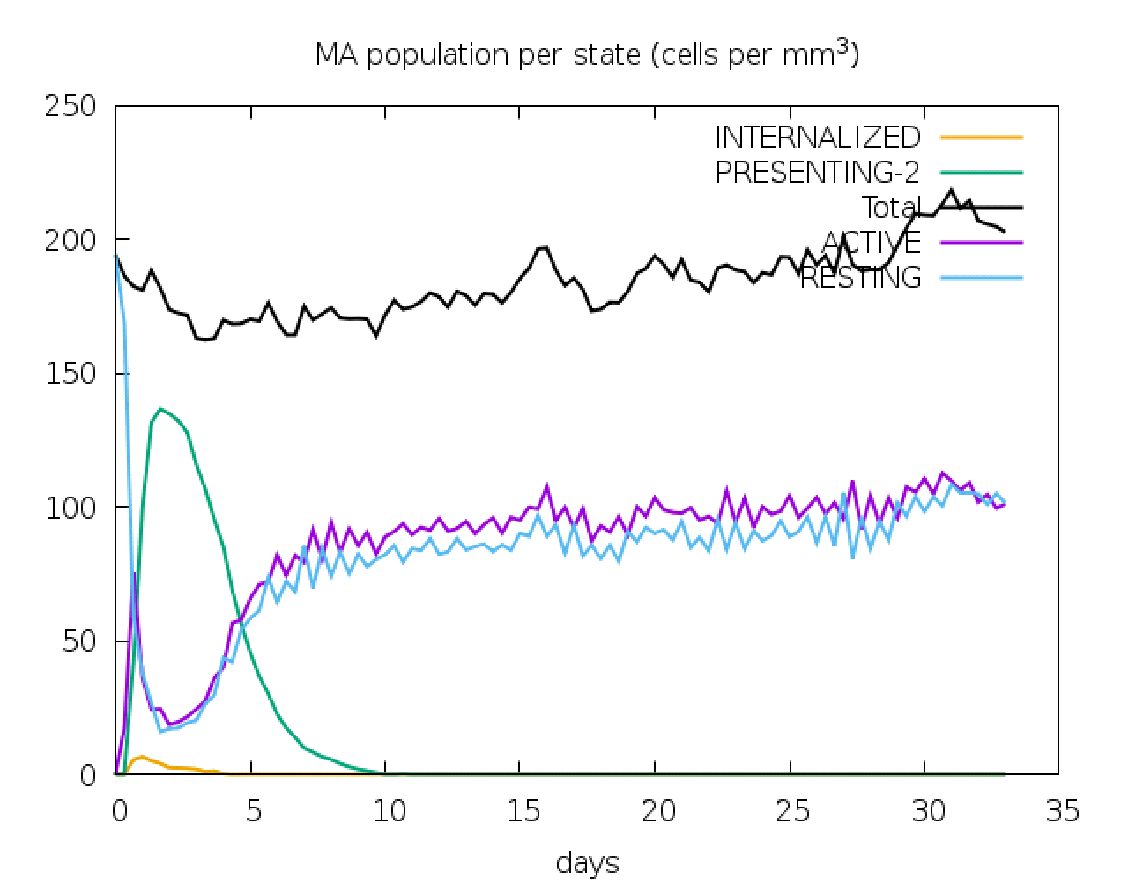

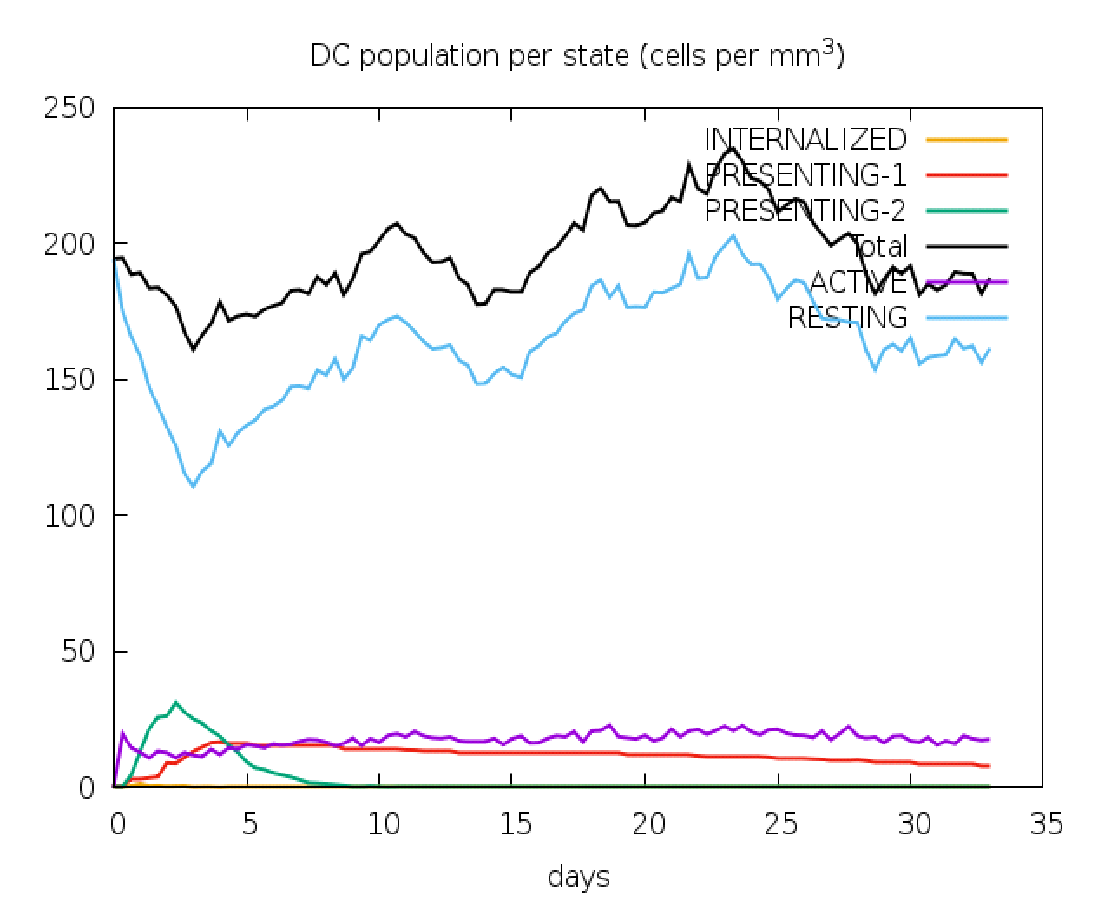


A B

2.6 : (A) Macrophages Total count, internalised, presentation on MHC class-II, active and resting macrophages (B) Dendritic cells. DC can present antigenic peptides on both MHC Class-I and class-II molecules. The curve shows the total number broken down to active, resting, internalised and presenting the age
